# Phenylsulfonimide PPARα Antagonists Enhance Nrf2 Activation and Promote Oxidative Stress-Induced Apoptosis/Pyroptosis in MCF7 Breast Cancer Cells

**DOI:** 10.3390/ijms24021316

**Published:** 2023-01-10

**Authors:** Marialucia Gallorini, Valentina Di Valerio, Isabella Bruno, Simone Carradori, Rosa Amoroso, Amelia Cataldi, Alessandra Ammazzalorso

**Affiliations:** 1 Department of Pharmacy, G. d’ Annunzio University, Via dei Vestini 31, 66100 Chieti, Italy; 2 Department of Medicine and Aging Sciences, G. d’ Annunzio University, Via dei Vestini 31, 66100 Chieti, Italy

**Keywords:** breast cancer, COX2, MCF7, Nrf2, oxidative stress, PPARα antagonist, pyroptosis, sulfonimides

## Abstract

The NF-E2-related factor 2 transcription factor (Nrf2) orchestrates the basal and stress-inducible activation of a vast array of antioxidant genes. A high amount of reactive oxygen species (ROS) promotes carcinogenesis in cells with defective redox-sensitive signaling factors such as Nrf2. In breast cancer (BC), emerging evidence indicates that increased Nrf2 activity enhances cell metastatic potential. An interconnection between peroxisome proliferator-activated receptors (PPARs) and Nrf2 pathways in cancer has been shown. In this light, newly synthesized PPARα antagonists, namely IB42, IB44, and IB66, were tested in the BC cell line MCF7 in parallel with GW6471 as the reference compound. Our results show that the most promising compound of this phenylsulfonimide series (IB66) is able to decrease MCF7 proliferation by blocking cells at the G2/M checkpoint. The underlying mechanism has been investigated, disclosing a caspase 3/Akt-dependent apoptotic/pyroptotic pathway induced by the increased generation of oxidative stress. Moreover, the involvement of Nrf2 and COX2 in IB66-treated MCF7 cell response has been highlighted. The reported data lay the groundwork for the development of alternative targeted therapy involving the Nrf2/PPARα molecular axis, able to overcome BC cell chemoresistance and cause better clinical outcomes, promoting other forms of programmed cell death, such as pyroptosis.

## 1. Introduction

The NF-E2-related factor 2 transcription factor (Nrf2) is widely recognized as the main intracellular defense mechanism, regulating the intracellular antioxidant pathway against exogenous and endogenous stimuli and thus being considered an important regulator of cell survival [1]. Accumulating evidence highlights that Nrf2 hyper-activation may play a direct role in the control of cell growth, being related to apoptosis [2] and oxidative stress-related pyroptosis, the inflammatory-related cell death [3]. Additionally, the pro-tumorigenic action of Nrf2 has been demonstrated in many tumors, by accelerating stress adaption, increasing drug resistance, and driving oncogenesis [4].

In breast cancer (BC), the role of Nrf2 in tumor growth is controversial and likely context-dependent [5]. However, emerging evidence indicates that increased Nrf2 activity may enhance the metastatic potential of BC cells. A recent study showed that the Nrf2 overexpression promotes the proliferation and migration of MCF7 and MDA-MB-231 breast cancer cells [6]. In addition, Nrf2 has been identified as a key regulator in the chemotherapeutic resistance of MCF7 cells under hypoxia conditions [7]. Considering this, targeting proteins which in turn can modulate the Nrf2-related pathway could represent a novel potential treatment for drug resistance in BC cells.

Peroxisome proliferator-activated receptors (PPARs) comprise three subtypes (PPARα, γ, and δ), and they belong to the superfamily of nuclear receptors. PPARs act as regulators of lipid metabolism, mitochondrial biogenesis, and antioxidant defense. Although their key role in regulating lipid metabolism has been well disclosed, the role of PPARs in regulating redox activity remains partially understood [8]. Different studies have shown an interconnection between PPARs and Nrf2 pathways. More in detail, both PPARα and PPARγ agonists were found to promote the Nrf2 activation to protect cells against oxidative stress [9,10]. It has been shown that mice treated with the PPARα agonist perfluorodecanoic acid overexpress hepatic β- and ω-oxidation enzymes, and their livers mount a compensatory hepatoprotective response via PPARα and Nrf2, markedly increasing hepatic efflux multidrug resistance-associated protein (Mrp) transporters [11].

The involvement of PPARs in lipid and glucose metabolism, cell differentiation, energetic homeostasis, immune, and chronic inflammatory responses make them attractive targets in disorders such as metabolic syndrome, diabetes, hypercholesterolemia, obesity, cancer, and other related conditions [12,13,14,15]. The close interplay between lipid oxidation, chronic inflammation, and cancer [16] oriented the researchers to also explore the antitumor activity of PPAR ligands, leading to a significant amount of literature data, sometimes reporting contrasting results. In fact, PPAR isoforms can function as tumor suppressors or inducers, depending on the biological context and the cancer type [17,18]. A remarkable attention has been paid to PPARα antagonists, whose activity on fatty acid metabolism could be responsible for a metabolic alteration of cancer cells, by promoting a switching from glycolysis to fatty acid oxidation [19]. Experimental evidence shows the antitumor potential for PPARα antagonists in glioblastoma [20], leukemia [21], renal cancer [22], pancreatic and colorectal cancer [23], head and neck paraganglioma [24,25], ovarian cancer, and melanoma [26]. Most important for this context, it has been shown that rat mammary gland epithelial cells showed significantly increased PPARα mRNA expression in carcinomas and PPARα ligand activation significantly increased the proliferation of MCF7 cells [27].

**Figure 1:**
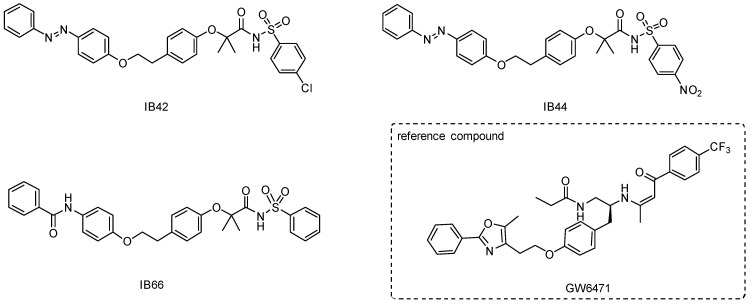
Chemical structures of phenylsulfonimide derivatives IB42, IB44, and IB66 tested in this study, and GW6471 used as reference PPARα antagonist.

In this light, the aim of this work was to deepen the knowledge about the possibility to interfere with BC cell growth and proliferation by targeting PPARα receptors. With this aim, PPARα antagonists were tested in the BC cell line MCF7. Our group has previously identified a family of sulfonimide and amide derivatives as selective and potent PPARα antagonists, displaying marked antiproliferative effects in the low micromolar range on several cancer cell lines (pancreatic, colorectal, and paraganglioma models) [25]. Sulfonimides represent interesting derivatives of sulfonamides, endowed with multiple biological activities; thanks to its properties, the sulfonimide functional group constitutes the scaffold of several compounds of pharmaceutical interest [28]. In this work, IB42 (hPPARα IC_50_ = 0.21 μM), IB44 (hPPARα IC_50_ = 1.1 μM), and IB66 (hPPARα IC_50_ = 0.24 μM) (Figure 1) were tested for their cytotoxic, pro-apoptotic, and anti-proliferative potential in human BC cells, also highlighting the possibility to interfere with the Nrf2 activity as oncogene and chemoresistance-associated proteins.

## 2. Results

### 2.1. Cell Metabolic Activity (MTT Assay) of MCF7 Exposed to Phenylsulfonimide Derivatives

**Figure 2:**
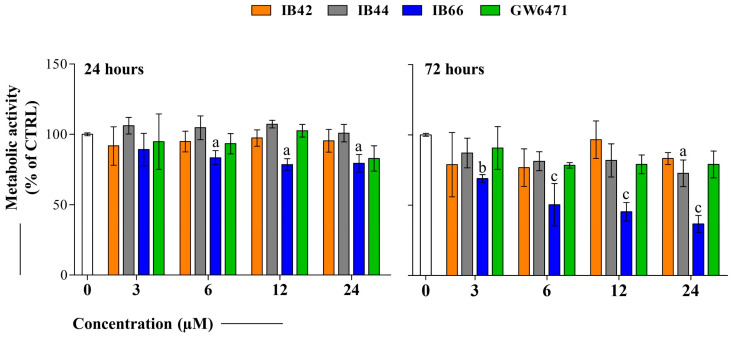
Cell metabolic activity of MCF7 cells in the presence of phenylsulfonimide derivatives. Bar graphs represent the cell percentage of metabolically active cells in the presence of increasing concentrations (0–3–6–12–24 µM) of IB42, IB44, IB66, and GW6471 after 24 and 72 h of exposure. Optical density readings in untreated cultures (0 µM) were set to 100%. Bars show mean values ± standard deviations summarized from individual values in independent experiments (n = 6). Lower case letters indicate significant differences between untreated cell cultures (0 µM) and treated samples: a (*p* < 0.01), b (*p* < 0.001), and c (*p* < 0.0001). CTRL = 0 µM.

The cell metabolic activity of the test compounds (3–24 µM) was measured by MTT assay after 24 and 72 h of exposure. The concentration range was chosen based on previous results obtained by testing the same compounds on several cancer cell lines and normal fibroblasts. In the 3–24 µM range, phenylsulfonimide derivatives did not affect cell viability of normal fibroblasts, thus disclosing selectivity on cancer cells [25]. After 24 h, IB42 only slightly decreased cell metabolism compared to the untreated sample, whereas IB44 was not effective at all the concentrations tested (Figure 2). On the contrary, IB66 significantly affected cell metabolic activity starting from 6 µM (83.4%). Interestingly, GW6471 weakly affected the MCF7 cell metabolism at all the concentrations tested. Again, when compounds were administered for 72 h, IB66 was the most effective in decreasing cell metabolic activity in the range tested, the percentages of active cells assessed being 68.9% at 3 µM and 36.6% at 24 µM.

### 2.2. Cytotoxicity Occurrence (LDH Release)

**Figure 3:**
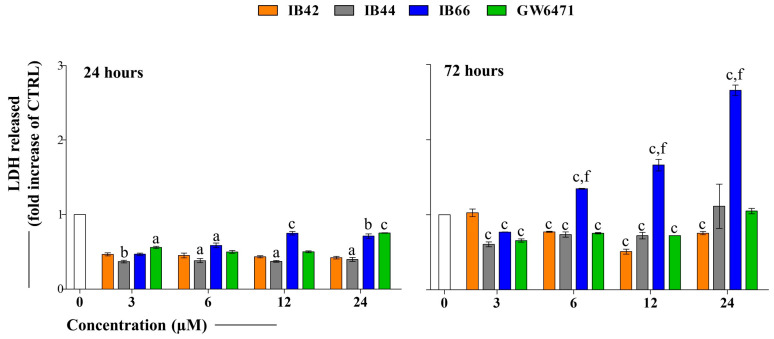
Cytotoxicity occurrence in MCF7 cells in the presence of phenylsulfonimide derivatives. Bar graphs present the LDH released from treated MCF7 cells as the fold increases in the one released from untreated cells after 24 and 72 h of exposure. Bars show mean values ± standard deviations summarized from individual values in independent experiments (n = 6). a (*p* < 0.01), b (*p* < 0.001), and c (*p* < 0.0001) between untreated cultures (0 µM) and treated samples. f (*p* < 0.0001) between GW6471-treated cultures and samples in the presence of phenylsulfonimide derivatives. CTRL = 0 µM.

The LDH release from MCF7 cells in the presence of the compounds was analyzed after 24 and 72 h (Figure 3). IB42 and IB44 were not effective under all the experimental conditions after 24 h of exposure. On the contrary, when IB66 and GW6471 were added to the cultures, the LDH was released in a dose-dependent manner, in amounts worth 0.46-fold and 0.7-fold of the untreated sample at 3 µM and at 24 µM, respectively. After 72 h of exposure, IB42 and IB44 maintained the 24 h trend, still not being effective regarding the LDH release.

On the contrary, IB66 was significantly effective, the release of LDH being almost doubled at 12 µM with respect to the untreated sample (1.66-fold) and almost tripled at 24 µM (2.66-fold). Interestingly, GW6471 did not increase cytotoxicity at 72 h. 

### 2.3. Cell Cycle Analysis

**Figure 4:**
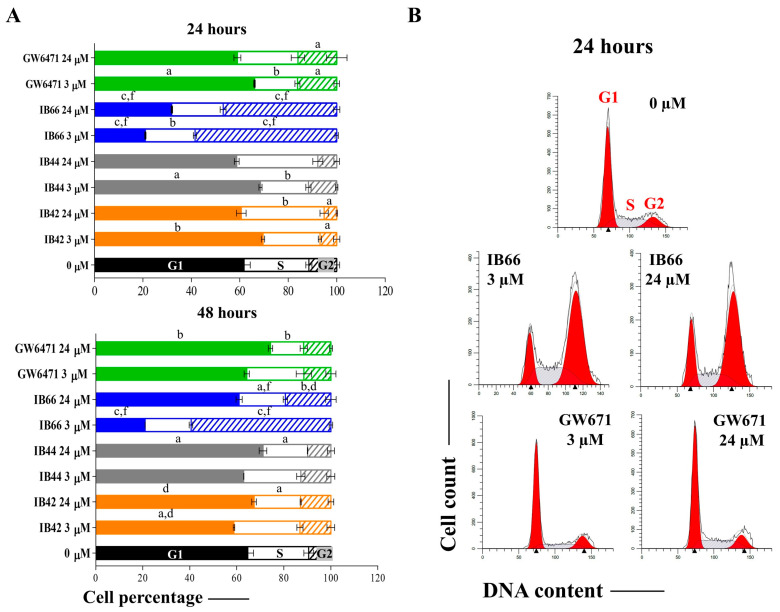
Cell cycle analysis in MCF7 cells in the presence of phenylsulfonimide derivatives. (**A**) The bar graph shows cell percentages in the various phases of cell cycle (G1, S, and G2) of MCF7 exposed to compounds at 3 and 24 µM after 24 and 48 h. Bars show mean values ± standard deviations summarized from individual values in independent experiments (n = 6). 0 µM = untreated control. a (*p* < 0.01), b (*p* < 0.001), and c (*p* < 0.0001) between untreated cultures (0 µM) and treated samples. d (*p* < 0.01) and f (*p* < 0.0001) between GW6471-treated cultures and samples in the presence of phenylsulfonimide derivatives. (**B**) Cell cycle profiles represented by fluorescence emission peaks obtained after the propidium iodide staining (*y*-axis = cell count; *x*-axis = propidium iodide fluorescence emission in the FL channel).

The cell cycle analysis was performed after 24 and 48 h in the presence of the lowest and the highest concentrations of compounds (3 and 24 µM, respectively) (Figure 4). After 24 h, cell percentages were assessed at 61.8% (G1 phase), 26.0% (S phase), and 11.2% (G2 phase) in the untreated sample (Figure 4A) and MCF7 cells were typically distributed in the various phases of cell cycle in the untreated sample (Figure 4B). In the presence of IB42 at 24 µM, an increase in cells in the S phase was registered (34.7%), while the G1 is diminished. Regarding IB44, an S-phase spread was registered but it was not significant with respect to the untreated sample. Contrariwise, in the presence of IB66 the MCF7 cell cycle profile was completely overturned with respect to the untreated control already at 3 µM. More in detail, the G1 phase dramatically decreased (20.95% at 3 µM and 31.9% at 24 µM) and the G2 phase significantly increased in parallel (58.7% at 3 µM and 46.8% at 24 µM). Notably, the cell cycle profile in the presence of the GW7461 antagonist was significantly perturbed but resulted to be more like the one of the untreated sample with respect to the one registered in the presence of IB66. After 48 h, the percentage trend was comparable to the one assessed at 24 h, except for the one in the presence of IB66 24 µM, which resembled the cell cycle profile of the untreated control, although the G2 phase remained significantly augmented.

### 2.4. Expression Levels of Apoptosis/Pyroptosis-Related Proteins

**Figure 5:**
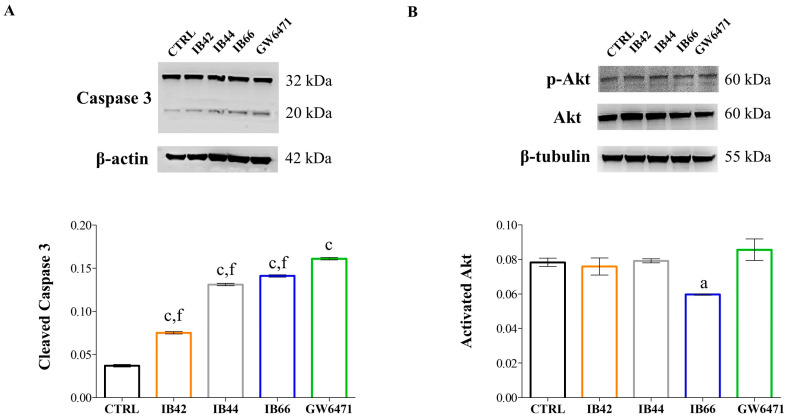
Apoptosis–pyroptosis occurrence in MCF7 cells in the presence of phenylsulfonimide derivatives at 3 µM after 24 h. β-actin and β-tubulin are used as loading controls. (**A**) Western blot analysis of caspase 3. 32 kDa = full-length protein; 20 kDa = cleaved caspase 3. Bar graph displays densitometric values related to cleaved caspase expressed as mean densitometric measurements ± S.D. (n = 3) normalized on the ones of the loading control and on the ones of the full-length protein. (**B**) Western blot analysis of activated Akt (p-Akt, phosphorylated protein). The bar graph shows densitometric values related to activated Akt expressed as mean densitometric measurements ± S.D. (n = 3) normalized on the ones of the loading control and on the ones of the non-phosphorylated protein. CTRL = untreated control. a (*p* < 0.01) and c (*p* < 0.0001) between untreated cultures (CTRL) and treated samples. f (*p* < 0.0001) between GW6471-treated cultures and samples in the presence of phenylsulfonimide derivatives.

The expression of cleaved caspase 3 and phosphorylated Akt (p-Akt) was quantified by Western blot analysis at 3 µM after 24 h of exposure (Figure 5). As for caspase 3 full length (32 kDa) in all the experimental conditions, fluctuations were hardly detectable as well as modifications of the expression of cleaved caspase 3 in the untreated sample (Figure 5A). Contrariwise, caspase 3 was significantly cleaved in all the other experimental conditions, to a major extent in the presence of IB44, IB66, and GW6471. 

In parallel, the ratio between p-Akt and Akt full length was not perturbed by IB42, IB44, and GW6471, while IB66 slightly but significantly decreased this ratio (Figure 5B).

### 2.5. Generation of Superoxide Anions

**Figure 6:**
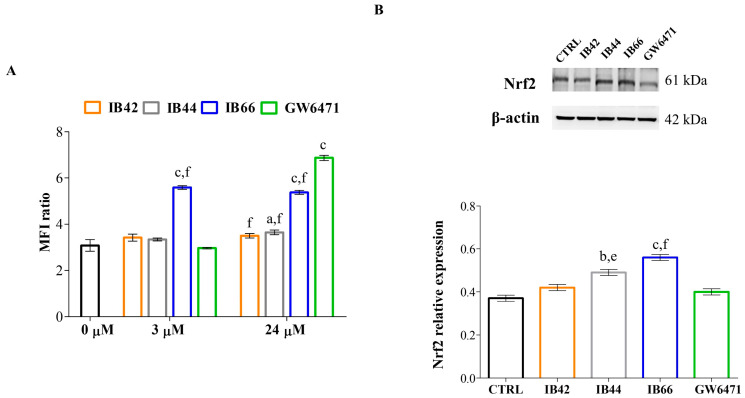
Generation of superoxide anions and Nrf2 expression levels. (**A**) The bar graph shows mean fluorescence intensity (MFI) ratios related to the emission in the FL-2 channel which are proportional to the generation of superoxide anions. Values are the mean ratios ± S.D. (n = 6) of the MFI generated from each sample on the unstained control (negative). 0 µM = untreated control. (**B**) Western blot analysis of Nrf2 in the presence of compounds at 3 µM for 24 h. β-actin is used as loading control. The bar graph shows densitometric values related to Nrf2 expressed as mean densitometric measurements ± S.D. (n = 3) normalized on the ones of the loading control. CTRL = untreated control. a (*p* < 0.01), b (*p* < 0.001), and c (*p* < 0.0001) between untreated cultures and treated samples. e (*p* < 0.001) and f (*p* < 0.0001) between GW6471-treated cultures and samples in the presence of phenylsulfonimide derivatives.

The formation of superoxide anions was quantified in MCF7 cells after 24 h of exposure at 3 and 24 µM (Figure 6A). The MFI ratio of the untreated cells was assessed at the amount worth 3.08-fold of the unstained sample. Among phenylsulfonimide derivatives, only IB66 was capable of significantly increasing the amount of superoxide anions (MFI ratio = 5.58) at 3 µM. Interestingly, GW6471 was not effective on the production of superoxide anions at the lowest concentration tested, whereas it was found to be the most effective one at 24 µM (MFI = 6.87) followed by IB66 (MFI = 5.37). 

### 2.6. Expression Levels of Oxidative Stress and Inflammation-Related Proteins

**Figure 7:**
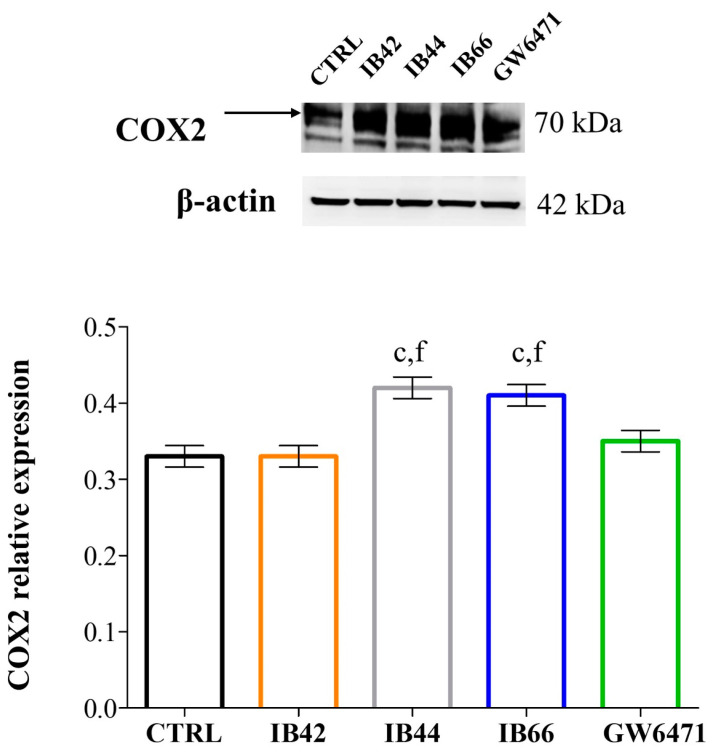
COX2 expression levels. Western blot analysis of COX2 in the presence of compounds at 3 µM for 24 h. β-actin is used as loading control. The bar graph shows densitometric values related to COX2 expressed as mean densitometric measurements ± S.D. (n = 3) normalized on the ones of the loading control. CTRL = untreated control. c (*p* < 0.0001) between untreated cultures and treated samples. f (*p* < 0.0001) between GW6471-treated cultures and samples in the presence of phenylsulfonimide derivatives.

The expression of the redox-sensitive protein Nrf2 (Figure 6B) and of the inflammation-induced COX or COX2 (Figure 7) was detected and quantified after 24 h at 3 µM. Among phenylsulfonimide derivatives, IB44 and even more IB66, caused the Nrf2 upregulation at the concentration administered, while the levels of expression in the presence of IB42 and GW6471 were comparable to the ones of the untreated sample. In the same experimental conditions, COX2 was upregulated by IB44 and IB66 as well. 

## 3. Discussion

BC is the most common malignancy in women, and one of the three most common cancers worldwide. Although therapy has progressed over the past years and early diagnosed BC is now considered potentially curable, there is still a need for newer targeted therapies which make the prospect of long-term disease control in metastatic BC an increasing reality [29]. Recently, it has been reported that redox-sensitive molecules such as Nrf2 may play a role in the control of BC progression [30] and the development of drug resistance and metastasis [31]. Additionally, an interplay between Nrf2-activated pathways and PPARs in BC has been shown. Nrf2 is an important player in the maintenance of mitochondrial homeostasis and structural integrity; under oxidative and inflammatory stress conditions, the protective role of Nrf2 is particularly critical [32]. The importance of mitochondrial metabolism in cancer survival and progression has recently been studied, suggesting that novel strategies targeting mitochondria could potentially represent the future of oncology [33]. Therefore, the interference of the Nrf2/PPAR signaling with mitochondrial functions during cancer cell apoptosis may represent a valuable anticancer strategy.

Interestingly, a recent study describes the synergistic anticancer effects observed in a BC model, induced by the combined treatment with anti-PD-L1 antibodies and the PPARγ antagonist GW9662 [34]. The increasing evidence of the anticancer effects played by PPAR antagonists could furnish novel therapeutic options, also in combination with traditional anticancer drugs and the emerging immunotherapy. The recent clinical development of TPST-1120, as the first-in-class PPARα antagonist as an anticancer drug, is noteworthy. Its anticancer activity involves the inhibition of tumor cell proliferation and angiogenesis, together with the stimulation of tumor-specific immunity. TPST-1120 is progressing into a Phase 1/1b study in combination with nivolumab (NCT03829436) and a randomized Phase 1b/2 study in combination with atezolizumab and bevacizumab in patients with advanced hepatocellular carcinoma (Tempest Therapeutics website, https://www.tempesttx.com, accessed on 19 December 2022).

In this context, three phenylsulfonimide derivatives targeting PPARα, namely IB42, IB44, and IB66, selected from a previous investigation [25], have been tested in vitro on human BC cells (MCF7 cell line) to verify their effectiveness as anti-proliferative and pro-apoptotic compounds. Moreover, the underlying molecular mechanism has been studied to verify whether oxidative stress occurrence may influence Nrf2 activation, and inflammation-related markers may be involved in their biological action.

Firstly, the biological effect of phenylsulfonimide derivatives was investigated in terms of the modulation of cell metabolic activity in comparison to the well-known PPARα antagonist GW6471 [35], disclosing a significant decrease in the presence of IB66 (Figure 2). To investigate whether the decrease in cell metabolic activity may be causally related to a cytotoxic event or to a block of cell proliferation, cytotoxicity occurrence and cell cycle progression were analyzed.

To assess cytotoxicity in MCF7 cells in the presence of phenylsulfonimide derivatives and GW6471, the LDH assay was performed in the same experimental conditions used for the MTT assay. Initially (24 h), the LDH was only slightly released in the presence of all the compounds with respect to the untreated sample (set as 1). After a longer exposure time (72 h), the cells dramatically released the LDH in the presence of IB66 in a dose-dependent manner. These data make it plausible to assume that MCF7 cells respond either weakly or not at all to the phenylsulfonimide derivatives IB42 and IB44, while IB66 is strongly effective. The decrease in cell metabolic activity could be related to the occurrence of cytotoxicity and cell necrosis, the permeabilization of plasma membrane being a key signature for necrotic cells, and this event can be quantified in tissue cultures by measuring the release of the enzyme LDH [36]. In order to assess whether the cytotoxic event may lead to an antiproliferative effect, a cell cycle analysis was performed after 24 and 48 h in the presence of the lowest and the highest concentrations of compounds (3 and 24 µM, respectively) (Figure 4).

The obtained data confirm the effectiveness of the IB66 compound on the metabolism and on the proliferation of MCF7 cells, but unexpectedly, disclose a molecular effect also for the two other phenylsulfonimide derivatives, IB42 and IB44. Since this effect does not seem to lead to a biological effect due to the results obtained from the MTT and the LDH assays, it is plausible to speculate a chemoresistance mechanism or a need of extended the exposure times to obtain an effect. Moreover, cell cycle analyses highlight that the three phenylsulfonimide derivatives exhibit a different behavior in terms of cell cycle perturbation. Indeed, the cells are blocked in the S phase in the presence of IB42 and IB44 as in the presence of GW6471, whereas the MCF7 cells accumulate in the G2/M phase in the presence of IB66. To induce a cell cycle arrest at the S phase can be compatible with an oxidative DNA damage caused by compounds, as already reported for MCF7 cells [37]. In parallel, the different cell behaviors in the presence of IB66 warrant in-depth exploration. In this light, molecules involved in the oxidative stress-induced apoptotic pathway were investigated.

Apoptosis has been recognized for a long time as the only mechanism capable of killing cancer cells by cytotoxic drugs. In recent years, studies have proved that pyroptosis, or inflammatory cell death, can also shrink tumors and inhibit cell proliferation [38]. Pyroptosis is oxidative stress-induced and Nrf2 has been reported to be tightly involved [39]. Moreover, pyroptosis has been recently investigated as a new therapeutic strategy against BC [40]. Both apoptosis and pyroptosis are caspase-dependent programmed cell death pathways and the cysteinyl aspartate specific proteinase-3 (Caspase-3) is a common key protein in both pathways [41]. Additionally, it has been reported that the induction of caspase 3-dependent pyroptosis is related to the inhibition of the PI3K/Akt pathway in cancer [42]. Against this background, caspase 3 and Akt expression levels were analyzed in MCF7 cells in the presence of phenylsulfonimide derivatives and GW6471 at 3 µM for 24 h (Figure 5). Additionally, the generation of superoxide anions and expression levels of Nrf2 were measured in the same experimental conditions (Figure 6). After 24 h of exposure, the levels of cleaved caspase 3 were found to be weakly but significantly increased in cells exposed to IB42 (Figure 5A). Notably, IB44, and even more IB66 and GW6471, can raise cleaved caspase 3 levels. In parallel, the expression levels of phosphorylated Akt (p-Akt) are found decreased only in the presence of IB66 (Figure 5B). Interestingly, superoxide anions are dramatically produced in the presence of IB66 already at the concentration of 3 µM, but not in the other experimental conditions (Figure 6A). At 24 µM, the levels of superoxide are maintained significantly high in the presence of IB66 and are also raised in MCF7 cells exposed to GW6471. As for Nrf2, all the phenylsulfonimide derivatives increase its expression, IB44, and even more IB66 (Figure 6B).

In our experimental model, the MCF cell death was therefore tightly related to oxidative stress occurrence and Nrf2 was one of the major players involved, as the expression levels of the inducible cyclooxygenase (COX2) were further quantified after 24 h at 3 µM (Figure 7). COX2 expression is associated with angiogenesis and lymph node metastasis in human BC, since prostaglandins increase the expression and activation of aromatase [43]. It has been widely reported that chemotherapy-induced COX2 upregulation by cancer cells defines their inflammatory properties and limits the efficacy of chemo-immunotherapy combinations [44]. As a matter of fact, under our experimental conditions, COX2 expression can also be detected in untreated MCF7 cells and is even more increased in all the experimental conditions. Although our data regarding COX2 confirm that its overexpression is a sign of chemoresistance, MCF7 cells essentially undergo cell death and cell cycle arrest after exposure to phenylsulfonimide derivatives, in particular in the presence of IB66. This experimental evidence makes it plausible to assume that MCF7 cells express chemoresistance through the overexpression of COX2, but phenylsulfonimide derivatives are able to trigger alternative and compensative molecular pathways, which lead to apoptosis/pyroptosis and cell cycle arrest.

## 4. Materials and Methods

### 4.1. Synthesis

The phenylsulfonimide derivatives IB42, IB44, and IB66 were synthesized, purified, and characterized as previously reported [25]. GW6471 was purchased from Cayman Chemical Company (Ann Arbor, MI, USA). Compounds were stored at −20 °C until the biological evaluation.

### 4.2. Cell Culture and Cell Exposure to Treatments

Human BC MCF7 (HTB-22™) cells were purchased from ATCC^®^ and maintained in Dulbecco’s Modified Eagle Medium (DMEM) high glucose supplemented with 10% of fetal bovine serum (FBS) and 1% of penicillin/streptomycin (all from Euroclone, Milan, Italy) at 37 °C and 5% CO_2_.

Cells were seeded according to the different experimental techniques and left to adhere for 24 h. Next, cells were exposed to increasing concentrations of compounds IB42, IB44, IB66, and GW6471 diluted in DMSO (final concentration lower than 0.1%). 

### 4.3. Cell Metabolic Activity (MTT Assay)

The cell metabolic activity of MCF7 human BC cells was assessed by the MTT (3–(4,5-dimethylthiazol-2-yl)-2,5-diphenyltetrazolium bromide) test (Sigma-Aldrich, Milan, Italy). Cells were seeded (0.1 × 10^4^/well) in a 96-well tissue culture-treated plate (Falcon^®^, Corning Incorporated, NY, USA) and left to adhere for 24 h. Next, cells were exposed to compounds IB42, IB44, IB66, and GW6471 (0–3–6–12–24 µM) for 24 and 72 h. After the exposure time, cells were incubated as previously reported [45].

### 4.4. Cytotoxicity Occurrence (LDH Assay)

After 24 and 72 h, cell supernatants were collected from the same plate used for the MTT assay, centrifuged at 450× *g* for 4 min and stored on ice. In order to quantify the cytotoxicity caused by compounds, the CytoTox 96^®^ Non-Radioactive Cytotoxicity Assay (Promega Corporation, WI, USA) was performed as already reported [45]. Values were normalized on the MTT test, and therefore the assessment of cytotoxicity was calculated according to the following formula: LDH released = (A − B)/C, with A = the O.D. of the LDH activity of sample, B = the O.D. of the blank sample, and C = the O.D. of the MTT assay. Results are expressed as the fold increase on the untreated sample set as 1.

### 4.5. Cell Cycle Analysis (Cell Proliferation)

MCF7 cells were seeded (0.115 × 10^5^/well) in a 12-well tissue culture-treated plate (Falcon^®^, Corning Incorporated, NY, USA) and left to adhere for 24 h. The growth medium was then removed, and cells were subsequently exposed to compounds (0–3–24 µM) up to 48 h. After the exposure times (24 and 48 h), cells were collected, and samples were prepared as reported elsewhere [45]. Samples were analyzed by a flow cytometer equipped with a 488 nm laser (CytoFlex flow cytometer, Beckman Coulter, CA, USA) in the FL-3 channel (620 nm of wavelength emission). The 2 × 10^4^ events/sample were collected and analyzed with CytExpert Software (Beckman Coulter, CA, USA). The percentages of cells in the G1, S, or G2 phases of the cell cycle were calculated after mathematical modeling of histograms using the ModFit LT™ software (Verity Software House, CA, USA).

### 4.6. Immunoblotting

Cells were seeded (0.3 × 10^5^/well) in a 6-well tissue culture-treated plate (Falcon^®^, Corning Incorporated, NY, USA) and left to adhere for 24 h. Next, MCF7 cells were exposed to compounds at the concentration of 3 µM for 24 h. Protein concentration was determined using a bicinchoninic acid assay (QuantiPro™ BCA Assay kit for 0.5–30 μg/mL protein, Sigma-Aldrich, Milan, Italy) following the manufacturer’s instructions as already reported [46].

The MCF7 cell lysates (20 μg/sample) were electrophoresed on a 4–20% SDS-PAGE Gel (ExpressPlus™ 10 × 8, GenScript Biotech Corporation, Nanjing, China) and transferred to nitrocellulose membranes. Nitrocellulose membranes were afterward blocked in 5% of non-fat milk or 5% of BSA, 10 mmol/L Tris pH 7.5, 100 mM NaCl, 0.1% Tween 20, and probed with the following primary antibodies: mouse anti-β-actin (Sigma-Aldrich, St. Louis, MO, USA) (dilution 1:10,000), rabbit monoclonal anti-Akt, anti-phospho-Akt and anti-COX2 (all purchased by Cell Signaling Technology, MA, USA) (dilution 1:1000), goat polyclonal anti-caspase 3 (dilution 1:200), and rabbit polyclonal anti-Nrf-2 (dilution 1:750) (all purchased by Santa Cruz Biotechnology, CA, USA). Next, membranes were incubated and immunoreactive bands were detected as described previously [46].

### 4.7. Detection of Mitochondrial Superoxide Anions

The intracellular generation of mitochondrial superoxide anions was determined after 24 h using the oxidation-sensitive fluorescent probe MitoSOX™ (MitoSOX™ Red Mitochondrial Superoxide Indicator, Invitrogen, Thermo Fisher Scientific, Waltham, MA, USA). MCF7 cells were seeded (0.115 × 10^5^/well) in a 12-well tissue culture-treated plate (Falcon^®^, Corning Incorporated, NY, USA) and left to adhere for 24 h. Then, growth medium was removed and replaced with fresh medium mixed with compounds (0–3–24 µM). After the exposure time, samples were prepared as reported elsewhere [47]. Cell suspensions were analyzed using a CytoFLEX flow cytometer (Beckman Coulter, Indianapolis, IN, USA) equipped with a 488 nm laser with the fluorescence channel FL-2/PE in a linear mode. Relative fluorescence emissions of gated cells by means of their forward and side scatter properties (FSC/SSC) were analyzed with the CytExpert software (Beckman Coulter, Indianapolis, IN, USA) and they were expressed as mean fluorescence intensity ratios on the unstained control (set as 1, not shown).

### 4.8. Statistics

Statistics were performed using the one-way analysis of variance (ANOVA) followed by the Tukey’s multiple comparison test by means of the Prism 5.0 software (GraphPad, San Diego, CA, USA). Results are the mean values ± standard deviations. Values of *p* ≤ 0.05 were considered statistically significant.

## 5. Conclusions

The design of innovative compounds targeting the Nrf2/PPARα molecular axis can be a strategy to overcome BC cell chemoresistance and invasiveness. In this scenario, the present study displays that the phenylsulfonimide PPARα antagonist IB66 is the most promising compound in terms of cytotoxicity occurrence and cell proliferation arrest at the G2/M checkpoint. IB66 increases caspase 3 cleavage, inhibits Akt phosphorylation, and increases superoxide anion generation, hallmarks of pyroptosis, an oxidative stress-related apoptosis. Additionally, IB66 modulates Nrf2 and COX2 expression levels. However, more in-depth and selective investigations, such as Nrf2 or PPARα knockout, are necessary to clarify the exact nature of the occurring cell death and will in general provide new insights about the molecular mechanism of action of IB66 in MCF7 cells. In conclusion, the increasing evidence of the anticancer effects played by PPAR antagonists could furnish novel therapeutic options, also in combination with traditional anticancer drugs and the emerging immunotherapy. 

## Data Availability

Data are contained within the article.
